# Neurological Improvement via Lysophosphatidic Acid Administration in a Rodent Model of Cardiac Arrest-Induced Brain Injury

**DOI:** 10.3390/ijms242417451

**Published:** 2023-12-14

**Authors:** Mitsuaki Nishikimi, Rishabh C. Choudhary, Muhammad Shoaib, Tsukasa Yagi, Lance B. Becker, Junhwan Kim

**Affiliations:** 1 Laboratory for Critical Care Physiology, Feinstein Institutes for Medical Research, Manhasset, NY 11030, USA; nishikim@hiroshima-u.ac.jp (M.N.); rchoudhary1@northwell.edu (R.C.C.); mshoaib1@pride.hofstra.edu (M.S.); tsuyagi-circ@umin.ac.jp (T.Y.); lance.becker@northwell.edu (L.B.B.); 2 Department of Emergency Medicine, North Shore University Hospital, Manhasset, NY 11030, USA; 3 Donald and Barbara Zucker School of Medicine at Hofstra/Northwell, Hempstead, NY 11549, USA

**Keywords:** biomarkers, lysophospholipids, cardiac arrest, brain function, neurological outcomes

## Abstract

Lysophosphatidic acid (LPA) serves as a fundamental constituent of phospholipids. While prior studies have shown detrimental effects of LPA in a range of pathological conditions, including brain ischemia, no studies have explored the impact of LPA in the context of cardiac arrest (CA). The aim of this study is to evaluate the effects of the intravenous administration of an LPA species containing oleic acid, LPA (18:1) on the neurological function of rats (male, Sprague Dawley) following 8 min of asphyxial CA. Baseline characteristics, including body weight, surgical procedure time, and vital signs before cardiac arrest, were similar between LPA (18:1)-treated (*n* = 10) and vehicle-treated (*n* = 10) groups. There was no statistically significant difference in 24 h survival between the two groups. However, LPA (18:1)-treated rats exhibited significantly improved neurological function at 24 h examination (LPA (18:1), 85.4% ± 3.1 vs. vehicle, 74.0% ± 3.3, *p* = 0.045). This difference was most apparent in the retention of coordination ability in the LPA (18:1) group (LPA (18:1), 71.9% ± 7.4 vs. vehicle, 25.0% ± 9.1, *p* < 0.001). Overall, LPA (18:1) administration in post-cardiac arrest rats significantly improved neurological function, especially coordination ability at 24 h after cardiac arrest. LPA (18:1) has the potential to serve as a novel therapeutic in cardiac arrest.

## 1. Introduction

Cardiac arrest (CA) is a major public problem that is responsible for over 300,000 deaths a year in the US. CA patients have a survival rate of approximately 10%, which has not been significantly improved for the last few decades. Even after successfully achieving the return of spontaneous circulation (ROSC), the majority of patients subsequently die, mainly due to post-CA brain damage. However, the precise mechanisms underlying this brain damage remain incompletely understood, and we lack effective therapeutic drugs, which has impeded the development of effective therapeutic options [1,2,3]. Therefore, the development of neuroprotective drugs is of great importance to enhance the survival rate of CA patients.

Lysophosphatidic acid (LPA), a phospholipid metabolite containing a single fatty acyl chain, serves as a potent signaling molecule participating in various critical cellular processes, such as cell proliferation, migration, and inflammation [4,5]. Research has consistently demonstrated a notable elevation in LPA concentrations within the plasma of patients afflicted by traumatic brain injury and nonvalvular atrial fibrillation [6,7]. Moreover, in a rat model of middle cerebral artery occlusion (MCAO), there was a noteworthy increase in LPA levels within the brain [8]. These findings suggest that LPA may have a significant role in the development of brain dysfunction.

More comprehensive in vitro investigations have revealed that LPA supplementation induces increased permeability in cultured brain microvascular endothelial cells [9,10,11] and leads to neuronal cell death [12]. In in vivo studies, intracerebroventricular or intravenous administration of LPA in an MCAO model resulted in an enlargement of cortical infarct size [8,13]. These studies emphasize the detrimental effects of LPA on the ischemic brain. However, it is noteworthy that these studies induced a rapid increase in local LPA concentration [8] or did not consider the different roles of LPA carrying different fatty acids. Notably, distinct fatty acids have been shown to have unique functional roles [14,15]. Therefore, the adverse effects of LPA may be specific to certain LPA species or evident at concentrations significantly surpassing physiological levels. Moreover, the influence of LPA on specific pathways or cellular processes may not necessarily correlate with its impact on overall organ function. Therefore, further research is necessary for a more comprehensive understanding of LPA’s effects on the brain in different pathologic conditions, with a particular emphasis on specific LPA species and their implications for systemic organ function.

In our present study, we investigated the impact of the intravenous administration of LPA-containing oleic acid, known as LPA (18:1), on neurological function using our established rat models of asphyxial CA. The selection of LPA (18:1) is based on the demonstrated neuroprotective effects of oleic acid against cerebral ischemia and its role in preserving cognitive functions [16,17], with cell toxicity observed only at concentrations significantly above physiological levels [18]. Additionally, the unique abundance of oleic acid in the brain underscores its significance in maintaining normal brain function [19]. We also assessed its influence on hemodynamics. Our findings revealed a substantial enhancement in brain function, thus demonstrating the beneficial effects of LPA (18:1) supplementation in the context of brain ischemia due to CA.

## 2. Results

### 2.1. Baseline Characteristics

**Figure 1:**
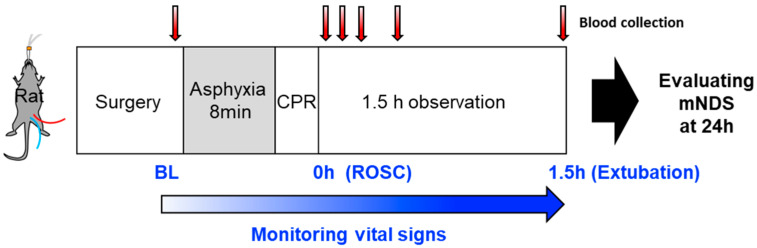
The experimental design and the timeline for blood sample collection and brain functional analysis. CPR; cardiopulmonary resuscitation, mNDS; modified neurological deficit scores, BL; baseline, ROSC; return of spontaneous circulation.

The experimental design and the timeline for blood sample collection and brain functional analysis are outlined in Figure 1. To minimize cardiac damage and its potential implications on brain function, we employed an 8 min mild cardiac arrest model [20]. On average, rats achieved ROSC within 1 min from the initiation of resuscitation and regained consciousness within 4 h after achieving ROSC.

### 2.2. Changes in LPA Levels Post-CA

**Figure 2:**
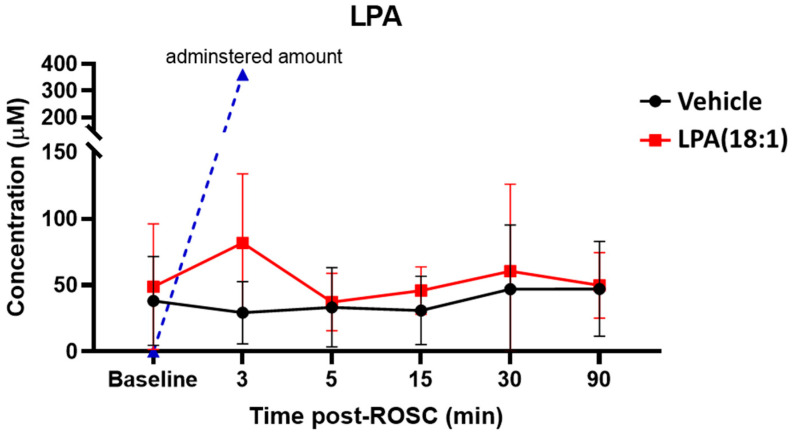
**Changes in total lysophosphatidic acid (LPA) levels in plasma after CA and with LPA (18:1) supplementation.** LPA concentrations do not change after CA (black). LPA levels after providing 10 mg/kg would be approximately 360 μM; however, post-CA plasma LPA levels only exhibit a slight increase after LPA administration and rapidly normalize to baseline levels (red). *n* = 2, ROSC; return of spontaneous circulation.

We first quantified alterations in plasma LPA levels following CA and with LPA (18:1) supplementation, as illustrated in Figure 2. Our observations revealed that total LPA levels remained relatively stable after CA for up to 90 min post-ROSC. Following the injection of LPC (18:1), LPA levels exhibited a noticeable increase at the 3 min mark after injection and rapidly returned to baseline levels within 5 min (Figure 2). Considering that the expected concentration of LPA after injecting 10 mg/kg of LPA (18:1) exceeds 300 µM, this small increase underscores the remarkably swift consumption of plasma LPA.

### 2.3. Effects of LPA (18:1) on Hemodynamics Post-CA

We subsequently evaluated the impact of LPA administration on hemodynamics by comparing blood pressure and heart rate between LPA (18:2)-treated rats and those treated with the vehicle. In both groups, baseline characteristics, including body weight, surgical procedure duration, vital signs before CA, time from asphyxia induction to CA, and the duration of cardiopulmonary resuscitation until ROSC, were similar, as outlined in Table 1.

**Figure 3:**
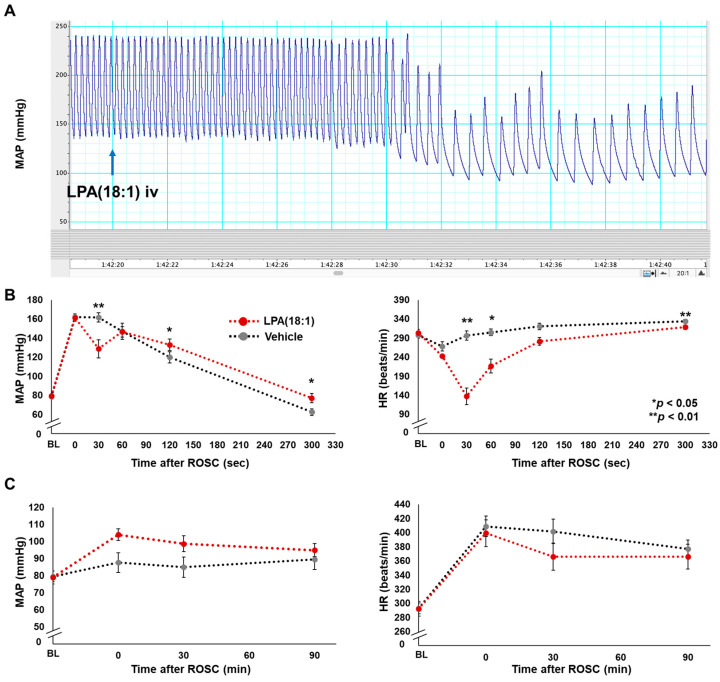
**Effects of lysophosphatidic acid (LPA) administration for blood pressure and heart rate post-cardiac arrest.** (**A**) Representative atrial pressure waveform shortly after LPA administration (iv), which was administrated approximately 1 min after the return of spontaneous circulation (ROSC). LPA iv typically induced transient bradycardia. The difference of changes in mean arterial blood pressure (MAP) and heart rate (HR) during short (**B**) and long (**C**) terms between LPA and vehicle groups. Each time point between the two groups was compared with a two-way repeated measures ANOVA. MAP; mean arterial blood pressure, HR; heart rate, BL; baseline.

Figure 3A shows a representative atrial pressure waveform shortly after LPA administration. Our investigation revealed that intravenous LPA administration induced transient bradycardia approximately 30 s after achieving ROSC. The distinctions in changes to mean arterial pressure (MAP) and heart rate (HR) over short and long durations between the LPA and vehicle intravenous (iv) groups are illustrated in Figure 3B,C, respectively. It is noteworthy that except for the transient decreases in both indices observed in the very early phase after ROSC in the LPA iv group, there were no statistically significant differences in the values of MAP and HR during the 90 min following ROSC, suggesting there is no difference in heart function between the LPA and vehicle iv groups. The data also suggest that alterations in brain function are not affected by improved brain perfusion.

### 2.4. Effects of LPA (18:1) on Brain Function Post-CA

**Figure 4:**
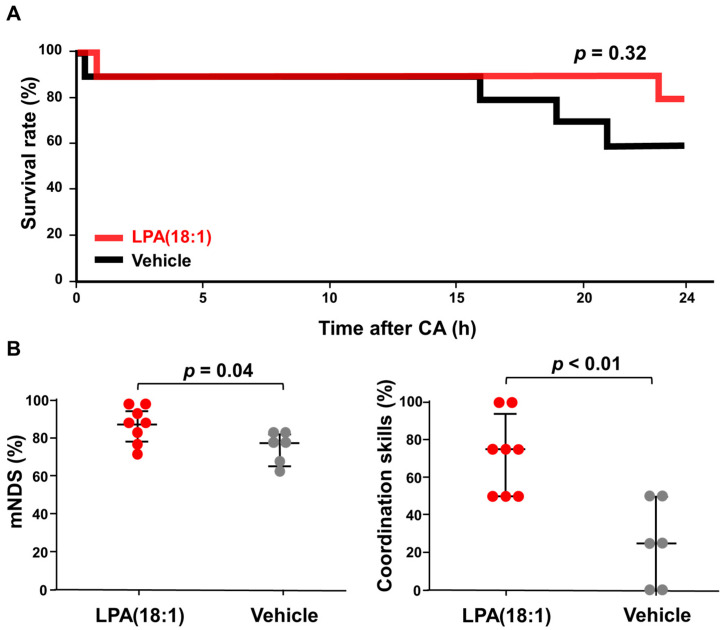
**Beneficial effect of lysophosphatidic acid (LPA) administration on rat survival and neurological function after 10 min of cardiac arrest (CA).** (**A**) Comparing survival rates between the LPA and vehicle groups for 24 h after cardiopulmonary resuscitation (*n* = 10 for each group). (**B**) Modified neurological deficit scores (mNDS) and the sub-points of the coordination skill in mNDS at 24 h after resuscitation for surviving rats in both groups. Differences between the groups were compared using the Mann–Whitney U test.

There was no statistically significant difference in the 24 h survival rates between the two groups, with 80% (8 out of 10) in the LPA (18:1) group and 60% (6 out of 10) in the vehicle group (Figure 4A). The Kaplan–Meier survival curve illustrates that LPA (18:1) administration did not yield a significant enhancement in survival outcomes (*p* = 0.32). However, we observed that the LPA-treated group exhibited improved neurological outcomes compared to the vehicle group (Figure 4B). Among the surviving rats, those treated with LPA (18:1) demonstrated notably higher overall modified neurological deficit scores (mNDS) at 24 h after cardiac arrest (LPA, 85.4% ± 3.1 vs. vehicle, 74.0% ± 3.3, *p* = 0.045). Notably, the most pronounced improvement was observed in the sub-parameter of coordination ability on the mNDS scores. The mean scores for the coordination ability sub-parameter were 71.9% ± 7.4 in the LPA group, and they were only 25.0% ± 9.1 in the vehicle group, signifying a statistically significant difference (*p* < 0.001). The data demonstrated that LPC (18:1) supplementation improved brain function after CA.

## 3. Discussion

In contrast to the adverse effects of LPA reported in prior studies, our research has demonstrated significantly improved brain function with LPA (18:1) supplementation following CA utilizing our established rat CA model. Particularly, we observed a significant improvement in coordination skills, underscoring the neuroprotective effects of LPA (18:1). The administration of LPA induced a brief episode of bradycardia in the early moments following the return of spontaneous circulation (ROSC). This observation aligns with a previous study indicating that LPA elicits vagal activation via the carotid body [21]. However, this bradycardic effect was short lived, lasting approximately 5 min after ROSC, and did not exert any detrimental effects on overall survival. While the precise implications of this transient bradycardia remain uncertain, our findings suggest that LPA (18:1) supplementation holds promise as a potential neuroprotective treatment in the context of CA.

**Scheme 1:**
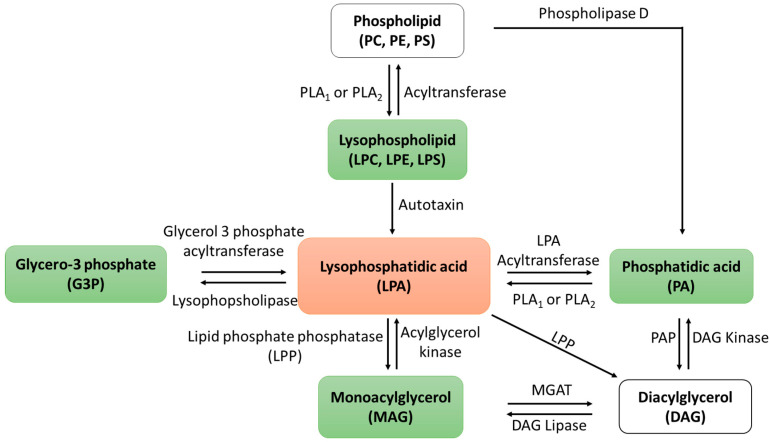
**Lysophosphatidic acid metabolic pathways.** The intricate metabolic pathways illustrate the rapid conversion of LPA into various lipid forms, underscoring the challenges in comprehensively studying the functional role of LPA.

The study of lipid functions, such as LPA, presents a significant challenge because these molecules do not exhibit well-defined regulatory functions as in enzymes. Instead, lipid metabolites are integrated into diverse metabolic pathways, the specifics of which may vary depending on the tissue type and underlying pathological conditions. Moreover, lipids possess the capacity to undergo facile conversions into different lipid types, introducing an additional layer of complexity when investigating their functions. The metabolic pathway of LPA, as elucidated in Scheme 1, greatly complicates the interpretation of the data at hand. For instance, any observed enhancement in function following autotoxin inhibition [22] could potentially result from the prevention of LPA generation or hydrolysis of lysophospholipids. Therefore, understanding the function of lipid metabolites may require the examination of its precursors as well as downstream metabolites of the lipid.

Another pivotal consideration in the study of LPA involves the specific fatty acids attached to it. Fatty acids linked to phospholipids are typically categorized into saturated fatty acids, monounsaturated fatty acids, and polyunsaturated fatty acids, each imparting distinct functional roles. For instance, previous research has highlighted the significance of linoleic acid in cardiac phospholipids [23], while this particular fatty acid exists in negligible amounts within the neuronal phospholipid pool [19,24]. Conversely, oleic acid is notably abundant in the brain relative to other tissues [19], and docosahexaenoic acid has been demonstrated as essential for normal brain function [25] as well as a protective factor for the brain during ischemia/reperfusion in numerous studies [26,27]. Notably, an increased incorporation of 22:6 in cardiac phospholipids has been associated with decreased cardiac mitochondrial function [19,24]. Therefore, it is reasonable to expect that LPAs containing different fatty acids may exert varying functional roles as shown in lysophosphatidylcholine [28]. However, it is worth noting that most studies exploring the role of LPAs have not explicitly specified the type of attached fatty acids [13], potentially contributing to discrepancies in the understanding of the neuroprotective capacities of different LPA species.

Due to the complex pathology of CA and the multifaceted functional roles of LPA (18:1), our study does not provide the neuroprotective mechanism of LPA (18:1). Additionally, our investigation did not encompass an assessment of long-term outcomes. However, despite these limitations, the enhancement in brain function and the observed trend toward improved survival with LPA (18:1) supplementation challenge the prevailing notion of adverse effects associated with LPA in the brain. Furthermore, our study also provides crucial factors considered for future studies examining the functional role of LPA.

## 4. Materials and Methods

### 4.1. Materials and Reagents

Reagent-grade chemicals were purchased from major commercial suppliers (Fisher Scientific, Hampton, NH, USA and Sigma Aldrich, St Louis, MO, USA). 1-Oleoyl-2-hydroxy-sn-glycero-3-phosphate (LPA (18:1)) was purchased from Avanti Polar Lipids (Alabaster, AL, USA). An LPA ELISA kit was purchased from Echelon Biosciences Inc. (Salt Lake City, UT, USA).

### 4.2. Animal Preparation

The Institutional Animal Care and Use Committees (IACUC) of the Feinstein Institutes for Medical Research approved the study protocol (IACUC protocol number 2017-033). Adult male Sprague Dawley rats (400–500 g, Charles River Laboratories, Wilmington, MA, USA) were used for this study. The procedures for asphyxia-induced CA and CPR were conducted as published previously [29]. Rats were anesthetized with 4% isoflurane (Isosthesia, Butler-Schein AHS Melville, NY, USA) and intubated with a 14-gauge plastic catheter (Surflo, Terumo Medical Corporation, Somerset, NJ, USA). Animals were mechanically ventilated with a fraction of inspired O_2_ (F_I_O_2_) of 0.3 and anesthesia was maintained with 2% isoflurane. The left femoral artery and vein were cannulated for monitoring arterial pressure and infusing medications, respectively. After the intravenous injection (iv) of heparin (300 U) and vecuronium bromide (2 mg/kg), asphyxia was induced by switching off the ventilator. CA, defined as a mean arterial pressure below 20 mmHg, was achieved within 3 min after the induction of asphyxia. After 8 min of asphyxia, mechanical ventilation was restarted at an F_I_O_2_ of 1.0, and chest compressions were performed at a rate of approximately 300 per min. At 30 s after the beginning of chest compressions, a 20 μg/kg bolus of epinephrine was administered, and chest compressions continued. Typically, rats achieve ROSC within 1 min after the initiation of chest compressions. Body temperature was controlled between 36 to 37 °C, and rats were then extubated 2 h after achieving ROSC.

### 4.3. LPA Administration

To test the beneficial effect of LPA administration, rats were randomized at a ratio of 1:1 into each group (*n* = 10). The 10 mg/kg LPA (18:1) mixed with 0.5% bovine serum albumin (BSA) in PBS (0.5 mL) was injected via the femoral vein cannula just after ROSC, with the vehicle group injected with only BSA. Survival was followed for 24 h with brain function evaluated in a blinded manner using the mNDS after resuscitation for surviving rats. The mNDS was expressed as a percentage, with 100% representing a perfect score [30]. The detailed scoring system is provided in Supplemental Table S1. The graphical illustration of the four coordination skills tests is shown in Supplemental Figure S1. Blood samples were collected at baseline and 3, 5, 15, 30, and 90 min after the injection of LPA (18:1). The concentration of LPA was measured as per the manufacturer’s protocol (Echelon Biosciences Inc., Salt Lake City, UT, USA).

### 4.4. Hemodynamics and Survival Outcomes

Hemodynamic monitoring was used to measure mean arterial pressure (MAP) and heart rate (HR). The time to reach CA was determined based on an MAP below 20 mmHg [31]. Death was confirmed by a mean arterial pressure below 30 mmHg lasting for 5 min while animals are under hemodynamic monitoring and by the absence of chest movement and sniffing for 5 min when animals are not under monitoring.

### 4.5. Statistical Analysis

Kaplan–Meier curves compared survival between the LPA and vehicle groups using the Wilcoxon test. The Mann–Whitney U test was used to compare two independent groups. A two-way repeated measures analysis of variance (ANOVA) was used to compare the vital signs between the two groups. Statistical analyses were performed using JMP software (version 17.0, SAS Institute Inc. Cary, NC). *p*-values of <0.05 were considered statistically significant. Data were expressed as the mean ± standard error of the mean.

## 5. Conclusions

Our study sheds light on the intricate nature of LPA and its potential as a neuroprotective agent following CA. While the multifaceted roles of LPA and its associated fatty acids add complexity to our understanding, our findings challenge the prevailing concept of adverse effects attributed to LPA in the brain. Notably, LPA (18:1) supplementation demonstrated improved coordination skills, showing its potential as a new neuroprotective drug in CA.

## Figures and Tables

**Table 1:** Baseline characteristics of the two groups for survival analysis.

Variable	Vehicle iv (*n* = 10)	18:1 LPA iv (*n* = 10)	*p*-Value
Body weight, g	436 ± 9.9	427 ± 8.4	0.79
Surgical procedure time, min	33.7 ± 1.9	32.6 ± 1.5	0.97
Before CA			
MAP, mmHg	76.6 ± 2.9	79.6 ± 3.4	0.85
HR, bpm	298 ± 7.3	293 ± 9.5	0.85
RR, times/min	45.8 ± 0.1	45.9 ± 0.1	0.54
BT, °C	36.3 ± 0.2	36.3 ± 0.1	0.76
Until CA time, s	111 ± 13.6	118 ± 11.7	0.82
CPR time, s	70 ± 3.8	69 ± 3.2	0.85

Data are presented as mean ± standard error. CA; cardiac arrest, MAP; mean arterial blood pressure, HR; heart rate, RR; respiratory rate, CPR; cardiopulmonary resuscitation.

## Data Availability

The data presented in this study are available in the article.

## Supplementary Materials

The following supporting information can be downloaded at https://www.mdpi.com/article/10.3390/ijms242417451/s1.
