# Supplementary material for: Neurological Improvement via Lysophosphatidic Acid Administration in a Rodent Model of Cardiac Arrest-Induced Brain Injury
Source: Int J Mol Sci. 2023 Dec 14;24(24):17451. doi: 10.3390/ijms242417451 (PMC10743439; doi:10.3390/ijms242417451)
Supplement: Supplementary file 1 [file ijms-24-17451-s001.zip › ijms-2718984-supplementary.pdf]

Supplementary to

Neurological Improvement via Lysophosphatidic acid Administration in a Rodent Model of Cardiac Arrest-Induced Brain Injury Supplementary to

Mitsuaki Nishikimi [1,2], Rishabh C. Choudhary [1,2], Muhammad Shoaib [1,3,] Tsukasa Yagi [1,2], Lance B. Becker [1-3], and Junhwan Kim [1-3,*]

Supplemental Table S1. Modified neurological deficit score (mNDS)

**mNDS**			
**1. General**			
Consciousness	Unresponsive (0), depressed (50), normal (100)		
Respiration	Abnormal (<60 or >120) (0), normal (100)		
		Total points	
**2. Cranial nerves**			
Olfactory	Orient to smell: no (0), yes (20)		
Vision	Visual stimulus startle response: no (0), yes (20)		
Corneal reflex	Blink response to corneal stimulus: no (0), yes (20)		
Whisker movement	Spontaneous: no (0), yes (20)		
Hearing	Startle response to loud noise: no (0), yes (20)		
		Total points	
**3. Motor**			
Left forepaw	Spontaneous or withdraw from pain: no (0), yes (10)		
Right forepaw	Spontaneous or withdraw from pain: no (0), yes (10)		
Left hindpaw	Spontaneous or withdraw from pain: no (0), yes (10)		
Right hindpaw	Spontaneous or withdraw from pain: no (0), yes (10)		
Tail	Spontaneous or withdraw from pain: no (0), yes (10)		
		Total points	/50
**4. Sensory**			
Left forepaw	React to pain: no (0), yes (10)		
Right forepaw	React to pain: no (0), yes (10)		
Left hindpaw	React to pain: no (0), yes (10)		
Right hindpaw	React to pain: no (0), yes (10)		
Tail	React to pain: no (0), yes (10)		
		Total points	/50
**5. Coordination**			
Ledge traverse	no (0), yes (25)		
Righting reflex	no (0), yes (25)		
Placing test	no (0), yes (25)		
Stop at table edge	no (0), yes (25)		
		Total points	
		**Total score**	
		**Percent NDS**	**(0-100%)**

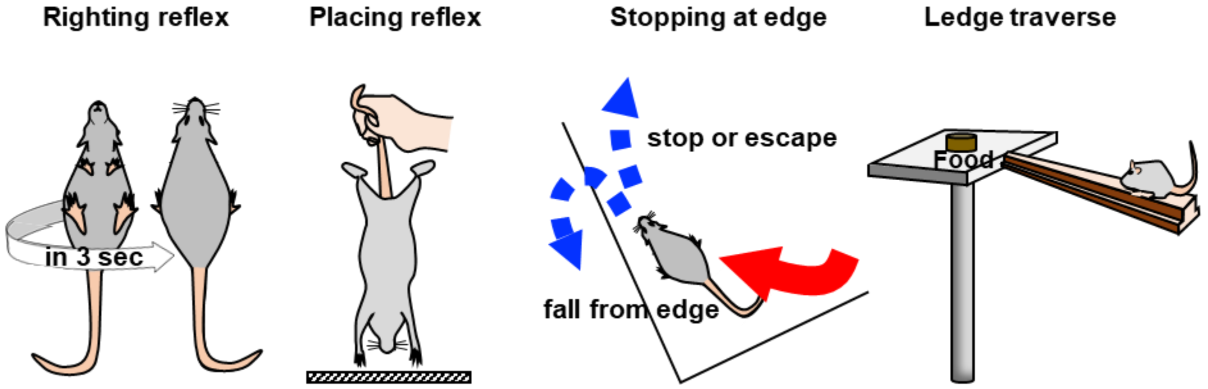

Supplemental Figure S1. Graphic illustration of four coordination skills tests.
